# The Impact of Nutritional Value on Consumer Attitudes and Repurchase Intentions: Price Fairness as a Moderator in the Cereal Market

**DOI:** 10.3390/foods14060938

**Published:** 2025-03-10

**Authors:** Min Gyung Kim, Joonho Moon

**Affiliations:** 1 College of Business Management, Hongik University, 2639, Sejong-ro, Jochiwon-eup, Sejong 30016, Republic of Korea; mkim@hongik.ac.kr; 2 Department of Tourism Administration, Kangwon National University, Chuncheon 24341, Republic of Korea

**Keywords:** cereal products, nutritional value, attitude, repurchase intention, and price fairness

## Abstract

The objective of this study is to explore the relationships among four key attributes—nutritional value, attitude, repurchase intention, and price fairness—in the context of the cereal product market. Additionally, the research investigates the moderating effect of price fairness on the relationship between nutritional value and consumer attitude using the low involvement theory as a theoretical underpinning. The study utilized the Clickworker platform to recruit 414 survey participants using an online survey, whose responses were analyzed using Hayes’ Process Macro Model 7. The findings reveal that nutritional value positively affects both consumer attitude and repurchase intention. Moreover, a significant positive relationship between attitude and repurchase intention was found. Importantly, price fairness was found to significantly moderate the relationship between nutritional value and consumer attitude, highlighting the role of perceived fairness in shaping consumer behavior. This research contributes to the literature by examining these relationships in the specific context of cereal products.

## 1. Introduction

**Figure 1:**
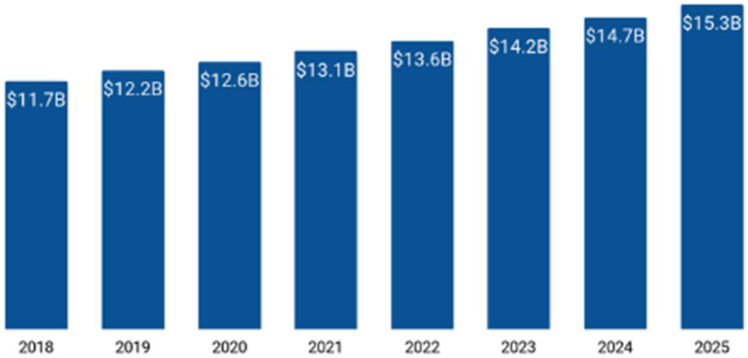
Cereal products of the current work. Sourced from https://www.t4.ai/industries/cereal-companies-market-share, accessed on 2 February 2025.

The global cereal market has experienced substantial growth in recent years. According to T4 [1], the demand increased from USD 11.7 billion in 2018 to USD 14.7 billion in 2024. The global market size for cereal products is depicted in Figure 1. According to Grand View Research [2], the market is experiencing a compound annual growth rate of 3.5%. These figures highlight the considerable prominence of the cereal market within the wider food industry. An in-depth understanding of consumer behavior is crucial for sustaining a competitive advantage in this highly competitive sector, which is primarily dominated by major industry players such as Quaker Oats, General Mills, Kellogg, and Post Holdings. This underscores the intense level of competition prevalent in the cereal market. Under this circumstance, it is essential to understand consumer characteristics to make strategies using constrained resources more efficiently. Given the motivation, this work is to investigate consumer behavior research in the area of cereal business.

**Figure 2:**
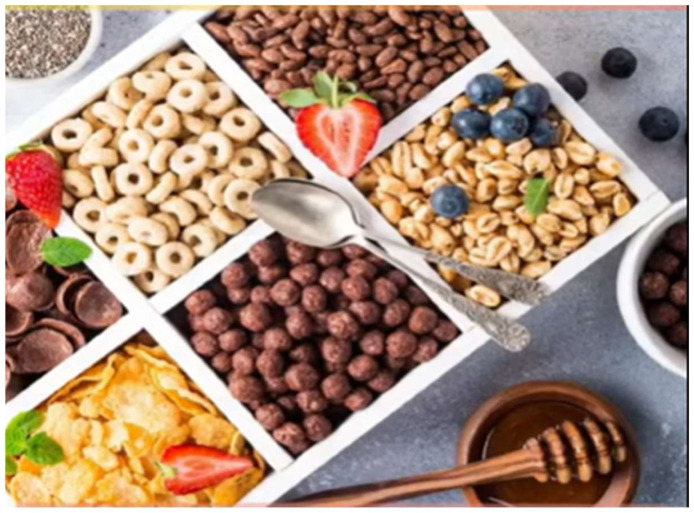
Cereal products of the current work. Sourced from https://www.youtube.com/shorts/fyRJqAEaQr4, accessed on 3 February 2025.

Figure 2 illustrates the specific cereal products examined in this study, which might include Cornflakes, Special K, Cheerios, Quaker Oats, Grape Nuts, etc. The research focuses on consumer behavior within the cereal market, particularly by exploring the relationships between nutritional value, consumer attitude, repurchase intention, and price fairness. Repurchase intention is chosen as the dependent variable because repeated purchases are closely linked to increased market share and sustained sales growth [3,4]. Nutritional value is identified as an important explanatory variable due to the growing consumer emphasis on health, driven by improvements in living standards and economic development [5,6]. As a result, nutritional value has become a key motivator for consumers seeking to enhance their health through dietary choices. Numerous studies have incorporated nutritional value as a central variable in understanding consumer preferences [5,7,8]. Numerous studies have emphasized nutritional value as a critical attribute in understanding consumer preferences [5,7,8]. Previous research indicates that cereal products exhibit certain nutritional deficiencies, which has prompted the introduction of products designed to address these concerns [9,10]. These claims indicate the significance of investigating the influence of the nutritional characteristics of cereal products on consumer behavior.

Consumer attitude is another crucial factor in this study, as it plays a significant role in shaping purchasing decisions. Attitude tends to be relatively stable and resistant to change, making it an important determinant of long-term consumer behavior [3,11]. Several prior studies have examined attitude as a key component in consumer behavior research [3,12,13], and this research aims to build on this literature by investigating the relationship between nutritional value, attitude, and repurchase intention.

This study incorporates price fairness, which refers to consumers’ perception of whether the price of a product is reasonable and justifiable [14,15]. Price fairness plays a significant role in determining price sensitivity and influencing consumer decision making. Price is a critical factor in consumer behavior, often regarded as a “pain point”, particularly in low-involvement product categories such as cereal [16,17]. Previous research has demonstrated that consumers in low-involvement product categories are particularly sensitive to price fairness [18,19,20]. Given this context, the low-involvement theory is adopted as the theoretical framework for examining consumer behavior. According to the low-involvement theory, consumer behavior and price sensitivity are influenced by whether a product is categorized as high or low [21,22,23]. Since cereal products are widely considered low-involvement goods [24,25], consumer behavior in this context will likely align with the principles outlined in the low-involvement theory. Therefore, understanding how perceived price fairness moderates the relationship between nutritional value and consumer attitude is crucial for explaining consumer behavior within the framework of the low-involvement theory. This relationship is particularly significant in the food sector, where price sensitivity and consumer attitudes are the key factors influencing purchasing decisions. While scholars argue that price is a critical determinant of consumer behavior [26,27], the extant literature has rarely explored cereal market behavior through the lens of the low-involvement theory focusing on nutritional cereal products. To streamline this gap, this work examines the moderating effect of price fairness on the relationship between nutritional value and attitude.

In summary, the main objectives of this study are twofold: (1) to examine the relationships between nutritional value, consumer attitude, and repurchase intention in the context of cereal products; (2) to explore the moderating role of price fairness in the relationship between nutritional value and attitude. This research, thus, makes a notable contribution to the literature by examining the dynamics between nutritional value, consumer attitude, and repurchase intention within the domain of cereal products. The study also offers theoretical insights by reinforcing the applicability of the low-involvement theory in the domain of cereal market consumers. Furthermore, it provides practical managerial recommendations to assist cereal manufacturers and marketers in making more informed decisions regarding product positioning and pricing strategies. Based on empirical findings, this study is likely to outline the key characteristics of consumer behavior related to the nutritional value, attitude, repurchase intention, and price of cereal products, while also suggesting areas for improvement in product factors associated with these elements.

## 2. Literature Review and Hypotheses Development

### 2.1. Nutritional Value

Nutritional value refers to the ability of specific foods to contribute to individual health by providing a balanced composition of essential nutrients, such as proteins, carbohydrates, and fats [5,7]. Scholars have further contended that nutritional value encompasses the content of these essential nutrients in food and beverages, which are vital for maintaining overall health, supporting growth, and supplying energy [7,8]. Biondi and Camanzi [28] posited that consumers are increasingly prioritizing the nutritional value of products, recognizing their direct link to health outcomes. In this context, Stelick et al. [8] asserted that nutritional value plays a crucial role in ensuring the sustainability of the food industry, as there is a growing awareness among consumers regarding the significance of a balanced, nutritious diet. According to Ali [29] and Sobaih et al. [6], nutritional content profoundly influences consumer behavior, particularly in response to the rising prevalence of lifestyle-related health conditions such as obesity, diabetes, cardiovascular disease, and hypertension. Moreover, Sadiq et al. [30] emphasized that foods with high nutritional value are instrumental in shaping positive consumer perceptions. Empirical research by Hati et al. [5] further demonstrated that nutritional value significantly influences consumer behavior, particularly in the context of frozen meat products. In conclusion, nutritional value is a pivotal factor driving consumers’ food choices and plays an essential role in shaping purchasing decisions.

### 2.2. Attitude

Previous studies have defined attitude as an evaluation of an object based on long-term perceptions, suggesting that attitudes are relatively stable and resistant to change [13,31]. Attitudes are generally regarded as psychological tendencies expressed through the evaluations of particular objects, products, services, or brands, whether positive or negative [32,33]. Consequently, scholars have extensively explored attitudes as a key attribute across various fields. Within the context of consumer behavior research, attitude is considered a crucial element, as it serves as a precursor to decision making and significantly influences the formation of a positive market reputation [3,11]. For example, Afrizal and Wallang [34] explored the users of electronic government services, positioning attitude as a central attribute. Similarly, Chawla and Joshi [35] focused on attitude when examining mobile wallet user behavior, and Jung et al. [13] employed attitude as a central variable in investigating consumer behavior within the apparel sector. Furthermore, attitude has been widely incorporated in studies addressing food product consumer behavior [3,11,12]. In particular, Ma and Chang [32] utilized attitude as a primary variable to examine consumer behavior toward environmentally friendly food products. Given the widespread application of attitude in consumer behavior research, it is clear that understanding and analyzing consumer attitudes provides valuable insights for predicting consumer behavior across diverse domains.

### 2.3. Repurchase Intention

Repurchase intention refers to consumers’ willingness to repeatedly purchase specific goods or services, reflecting their intent to continue engaging with a product or brand based on prior experiences [3,4]. Scholars identified repurchase intention as a crucial indicator of customer loyalty and satisfaction, demonstrating how positively consumers perceive a product or brand, which in turn influences their future purchasing decisions [36,37]. Previous research emphasizes the critical role of repurchase intention for businesses aiming to achieve stable and consistent sales growth [11,38]. Given its direct relationship with revenue expansion, repurchase intention has emerged as a fundamental construct in consumer behavior studies [11,39]. For instance, Hussain et al. [38] investigated repurchase intention as a central factor in the context of fashion products, while Loh and Hassan [40] explored it as a dependent variable to understand consumer behavior toward food truck offerings. Also, Anshu et al. [11] examined the determinants of repurchase intention within grocery retailing. Prior studies have also incorporated repurchase intention as the dependent variable in research on food delivery applications [41], food trucks [37], and consumer behavior toward green agricultural foods [36]. Collectively, these studies underscore repurchase intention as a well-established and extensively examined concept within the consumer behavior literature.

### 2.4. Hypotheses Development

Sadiq et al. [30] argued that consumers consider nutritional value a critical factor in food assessment given the strong connection between food and its potential to either promote or undermine health. In a similar vein, Park and Namkung [42] documented that the nutritional benefits of food, particularly those that promote individual health, play an essential role in shaping positive attitudes toward food. This suggests that the nutritional attributes of food significantly influence consumer evaluations, a concept that is particularly relevant to cereal products. Thanki et al. [43] also noted that nutritional motivations, which contribute to improved health, serve as key drivers in fostering both positive attitudes and repurchase intentions among organic food consumers. In a related study, Su et al. [44] identified a positive association between the nutritional value of milk and consumer attitudes, while Baiano [45] observed a similar positive effect of nutritional value on attitudes toward 3D-printed food. Ali [29] demonstrated that consumers tend to hold favorable attitudes toward food products that promote health through the use of fresh ingredients. Similarly, Hati et al. [5] showed that nutritional value positively influences consumer purchase intentions for food products. Lin et al. [7] also highlighted the positive impact of nutritional value on the repurchase intention of fresh food. Xu et al. [36] further found a positive relationship between nutritional value and repurchase intention for agricultural food products. Based on these findings, we propose the following research hypotheses:

**Hypothesis** **1:** *Nutritional value exerts a positive influence on attitude to cereal products*.

**Hypothesis** **2:** *Nutritional value exerts a positive influence on the repurchase intention of cereal products*.

Chawla and Joshi [35] emphasized the critical role of attitude in consumer behavior research, highlighting that attitude acts as an antecedent to loyalty, which subsequently drives business sales growth. Scholars have consistently explored the positive impact of attitude on repurchase intention, particularly within the food sector [11,12]. Hussain et al. [38] investigated halal cosmetic consumers and found that attitude significantly contributes to fostering higher levels of repurchase intention. Similarly, Asti et al. [3] revealed a positive relationship between attitude and repurchase intention in the context of e-grocery shopping. Thanki et al. [42] examined organic food consumers and demonstrated that attitude played a significant role in enhancing repurchase intention. Moreover, Nazri et al. [37] demonstrated the positive impact of attitude on repurchase intention within the food truck sector. From the review of the literature, the current study proposes the following research hypothesis:

**Hypothesis** **3:** *Attitude exerts a positive influence on the repurchase intention of cereal products*.

### 2.5. Moderating Roles of Price Fairness Based on Low-Involvement Theory

Price fairness refers to how consumers assess the price of a product in relation to their expectations and the perceived value it provides [16,17]. Consumers perceive price fairness when the price is deemed reasonable or affordable [15,46]. Conversely, price fairness is perceived as poor when the goods or services are priced too high, leading to greater consumer dissatisfaction and a stronger sense of price-related discomfort [14,19]. Price fairness has been widely explored within the food market, where it is considered an indirect measure of price sensitivity [14,17,46]. Moreover, consumers tend to have higher expectations for more expensive goods, expecting greater value or higher quality in exchange for the elevated cost [15,19]. This heightened expectation suggests that price-sensitive consumers are more likely to seek additional benefits from suppliers when faced with higher prices.

The low-involvement theory provides a solid theoretical framework for understanding consumer behavior in relation to low-involvement products. According to this theory, low-involvement products are generally characterized by frequent repurchases, relatively low prices, and low-risk decision making [21,22,47]. Researchers have argued that consumers of low-involvement products are particularly price-sensitive, with promotional strategies such as price discounts playing a crucial role in driving sales [21,47,48]. In the context of cereal products, consumers who perceive poor price fairness may expect additional benefits, as their expectations tend to rise in proportion to the cost of the product. Moreover, compared to other food categories like meat or fruits, cereal products are relatively low-cost, which typically makes consumers more sensitive to price changes [24,25]. Consumers of low-involvement products typically base their decisions on limited information, often forgoing extensive research [49,50]. In contrast, high-involvement consumers tend to engage in detailed information searches and make decisions after careful consideration, which is common for more expensive products [51,52]. These consumers also tend to adopt a long-term perspective, investing considerable time in gathering information before making a purchase [52,53].

In the context of price fairness, previous works noted that sharp price increases can negatively affect consumers’ perceptions of fairness [54,55]. When consumers perceive price unfairness, they are likely to become more sensitive to other product attributes, such as nutritional value. Given the relatively low cost of cereal products, even small price increases can provoke strong consumer reactions, significantly influencing their overall product evaluation. By integrating these findings, it becomes clear that consumer perceptions of cereal products are closely tied to their views on price fairness. Heightened sensitivity to price changes can, therefore, influence product appraisal in the case of nutritional cereal products. Furthermore, cereal products have long faced criticism for their nutritional aspects, particularly their high sugar content. In consequence, products have been introduced to address these concerns [9,10]. Given these factors, it is crucial to examine how both nutritional and price attributes influence consumer behavior and product evaluation. Considering both aspects together will offer valuable insights into the key drivers of consumer decision making in the cereal market. This study, therefore, proposes the following research hypothesis:

**Hypothesis** **4:** *Price fairness significantly moderates the relationship between nutritional value and attitude to cereal products*.

## 3. Method

### 3.1. Research Model

**Figure 3:**
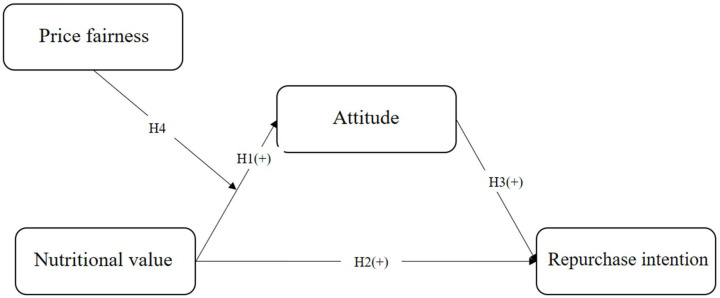
Research model.

Figure 3 illustrates the research model. Nutritional value positively influences both attitude and repurchase intention, with attitude also having a positive effect on repurchase intention. Also, price fairness moderates the relationship between nutritional value and attitude.

### 3.2. Measurement

Table 1 describes the measurement items. This research referenced previous works to derive the measurement items for the attributes: price fairness [15,45], nutritional value [5,7], attitude [3,13], and repurchase intention [3,4]. Also, this work adjusted items to make them more suitable for the current work. Regarding the operational definition, price fairness is how consumers evaluate the price of cereal rationally, and nutritional value is how individuals appraise the ingredients of cereal that are useful to promote health conditions. Moreover, the definition of attitude is the long-time-based assessment of cereal products. Last, the definition of repurchase intention is how individuals intend to buy cereal products again. Each variable was measured using four items. The survey also collected demographic information, including sex (0 = male, 1 = female), age (1 = 20–29 years old, 5 = older than 60 years old), weekly eating frequency (1 = less than once, 4 = every day), highest level of education (1 = less than a bachelor’s degree, 3 = graduate degree), and monthly household income (1 = less than USD 2500, 5 = more than USD 10,000).

### 3.3. Recruitment of Survey Participants

This study employed the Clickworker system service (https://www.clickworker.com/, accessed on 17 September 2024) for data collection. Previous research has successfully utilized Clickworker as a data collection tool [4,56,57]. Its widespread use motivated the adoption of this system for the present study because the data quality could be assured by the prior works for statistical inference. The data collection period spanned from 17 September to 2 October 2024, resulting in a total of 414 valid observations. Also, this study collected survey responses using an online platform and provided monetary compensation to the respondents in exchange for encouraging their participation. Data were gathered from participants in the United States and Canada, as cereal products are popular in North America. This regional focus was expected to yield more relevant insights based on the participants’ experiences with the product. Because Clickworker allows researchers to access the panels in the North American market, this work employed the system for the data collection. Moreover, this study employed a random sampling method to collect survey responses from a diverse group of individuals online. This approach was chosen because cereal products are widely accessible to everyone, and collecting random responses is likely to more accurately reflect the market’s reactions.

Table 2 provides detailed information about the survey participants. The sample comprised 70% males. Age distribution was as follows: 68 participants in their 20s, 129 in their 30s, 150 in their 40s, 56 in their 50s, and 11 aged 60 or older. Regarding educational background, 159 participants had less than a bachelor’s degree, while 174 held a bachelor’s degree. In terms of monthly household income, approximately 66.4% of the participants reported a household income of less than USD 5000.

### 3.4. Data Analysis

This study conducted frequency analysis to obtain demographic information from survey participants. To assess the goodness of fit, the following criteria were applied: a Kaiser–Meyer–Olkin (KMO) measure of sampling adequacy greater than 0.7, and Bartlett’s test of sphericity (χ^2^) [58]. Furthermore, in the argument of Hair et al. [57], the following standards were used to evaluate the convergent validity of the measurement items: factor loadings greater than 0.5, Cronbach’s α greater than 0.7, and eigenvalues greater than 1. A correlation analysis was performed to examine the relationships among the key variables: nutrition value, attitude, repurchase intention, and price fairness. This work also calculated the means and standard deviations for each. Path analysis was conducted using Hayes’ Process Macro Model 7, which utilizes ordinary least squares (OLS) regression. According to Hayes [59], Hayes’ Process Macro is advantageous in minimizing sample distortion and providing more robust estimations, as it is less influenced by the normality of the data. Also, Hayes [59] documented that Hayes’ Process Macro enables researchers to simultaneously analyze complex mediation and moderation models for estimation. Furthermore, a median split analysis was employed to examine the moderating effect of nutritional disclosure. The median split values for the relevant variables were as follows: nutritional value = 3 and price fairness = 3. To test the research hypotheses, we further implemented Hayes’ PROCESS Macro Model 7 with bootstrapping (5000 resamples). Finally, a median split analysis was conducted to explore the moderating effect of review information by constructing four distinct groups based on combinations of nutritional value and price fairness: (1) low nutritional value × low price fairness, (2) low nutritional value × high price fairness, (3) high nutritional value × low price fairness, and (4) high nutritional value × high price fairness. Furthermore, this work implemented regression analysis twice—once including control variables and once excluding them—to ensure the robustness of the estimations. The control variables consisted of demographic information. A simple slope analysis was additionally performed to examine differences in the slope across low, medium, and high price fairness groups.

## 4. Results

### 4.1. Validity and Reliability of Measurement Items and Correlation Matrix

Table 3 displays the results of the factor analysis. The model is statistically significant, as indicated by the Kaiser–Meyer–Olkin (KMO) measure of sampling adequacy (0.909) and Bartlett’s test of sphericity (Approx. Chi-Square, *p* < 0.05). All the factor loadings and Cronbach’s α values exceed the acceptable thresholds, confirming the reliability and validity of the measurement model. Based on these results, this study identified four key attributes: price fairness, nutritional value, attitude, and repurchase intention. Table 2 shows the mean values for each attribute: price fairness (mean = 3.04), nutritional value (mean = 3.18), attitude (mean = 4.23), and repurchase intention (mean = 4.38).

This research presents the correlation matrix in Table 4. Repurchase intention positively correlates with attitude (r = 0.654, *p* < 0.05), nutritional value (r = 0.417, *p* < 0.05), and price fairness (r = 0.264, *p* < 0.05). Attitude positively correlates with nutritional value (r = 0.554, *p* < 0.05) and price fairness (r = 0.322, *p* < 0.05). Nutritional value positively correlates with price fairness (r = 0.425, *p* < 0.05).

### 4.2. Results of Hypotheses Testing

Table 5 displays the results of Hayes Process Macro Model 7. All the models are statistically significant based on the *p*-values of F-value (*p* < 0.05). The results showed that the nutritional value positively affected attitude (β = 1.070, *p* < 0.05). The moderating effect of price fairness between the nutritional value and attitude appeared to be significant (β = −0.071, *p* < 0.05). The results also indicated that price fairness exerted a positive effect on attitude (β = 0.393, *p* < 0.05). Repurchase intention is positively influenced by the nutritional value (β = 0.066, *p* < 0.1) and attitude (β = 0.350, *p* < 0.05).

**Figure 4:**
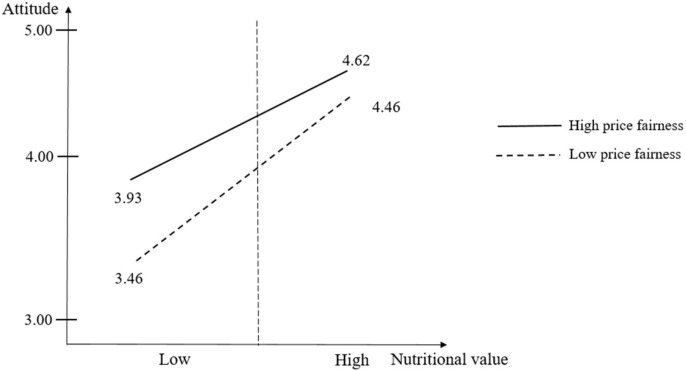
Moderating effect of price fairness on the relationship between the nutritional value and attitude using the median split analysis.

Figure 4 exhibits the results of the median split analysis. The results revealed that the difference between the low nutritional value and high nutritional value groups was 1.00 for the high price fairness group. The difference between the low nutritional value and high nutritional value groups was 0.69 in the case of the high price fairness group. It indicates that the low price fairness group is more sensitive to the nutritional value for building attitude.

**Figure 5:**
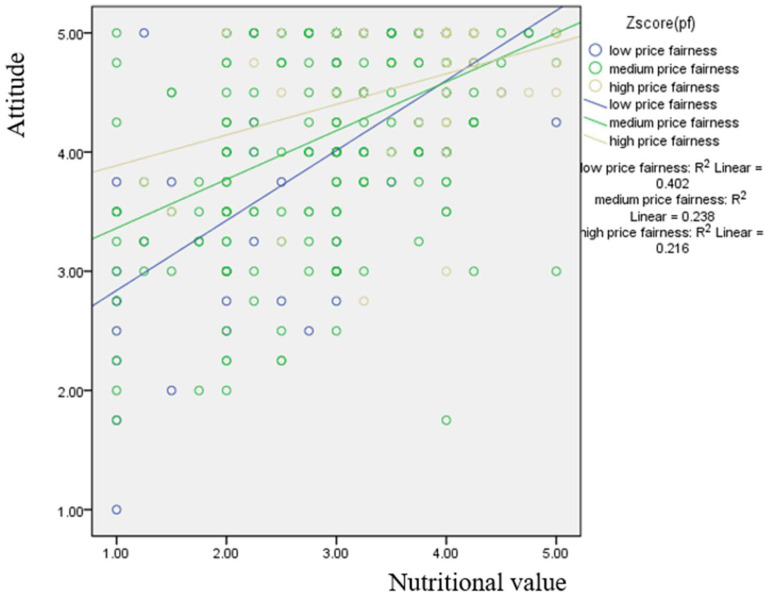
Moderating effect of price fairness on the relationship between the nutritional value and attitude using the simple slope method.

Figure 5 presents the results of simple. The results indicate that the group with low price fairness shows the steepest effect, while the group with high price fairness exhibits the most gradual effect. In detail, price-sensitive consumers are more sensitive to the effect of nutritional value on attitude. However, consumers less concerned about price are less sensitive to the impact of nutritional value on their attitude toward cereal products.

## 5. Discussion

This research investigated the consumers of cereal products using a survey focusing on the North American market. This work recruited the survey participants using Clickworker platforms and 414 observations were used to analyze the data. The mean values noted that consumers assess the price of cereal products a little bit skeptically because its mean value was the lowest (mean = 3.04). Also, the mean value of nutritional value presented that consumers evaluate the nutritional aspect of cereal products as somewhat insufficient (mean = 3.18). The mean value of repurchase intentions (mean = 4.38) was relatively high, likely due to the possibility that cereal products are commonly consumed in daily life.

**Figure 6:**
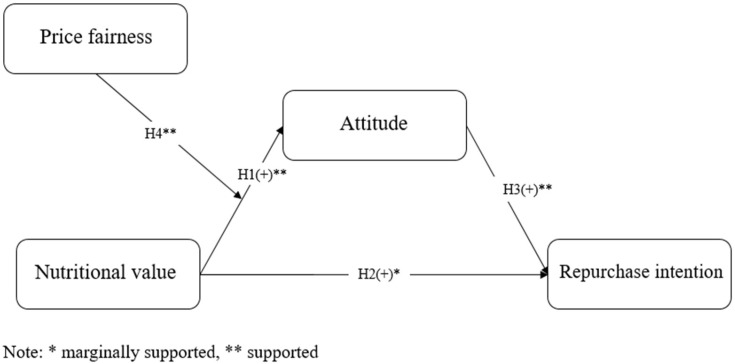
Results of hypotheses testing.

This study employed hypothesis testing using Hayes’ Process Macro Model 7. The results are summarized in Figure 6. The findings reveal a positive effect of nutritional value on both consumer attitude and repurchase intention. Consumer attitude toward cereal products also positively influenced repurchase intention. These results confirmed that nutritional value plays a critical role in shaping positive consumer attitudes and increasing repurchase intentions. This work further demonstrated that favorable attitudes toward cereal products led to a higher likelihood of repurchasing cereal products. These findings are aligned with previous research on the relationship between nutritional value, attitude, and repurchase intention in the context of food products [36,37]. It indicates that insights from consumer behavior studies in the food sector are applicable to cereal product consumers.

Although cereal products are often considered low-involvement items with limited nutritional value, previous studies have inadequately explored consumer behavior using the low-involvement theory, specifically within the context of cereal products. To minimize such a research gap, this work inspected the moderating role of price fairness in the relationship between nutritional value and consumer attitude. The study focused on the idea that high price fairness is associated with inexpensive, affordable, and reasonable pricing. The moderating effect of price fairness was negative and significant based on the low-involvement theory as the theoretical foundation. This implies that consumers tend to be more price-sensitive when purchasing low-involvement products, such as cereal. With health-related attributes playing an increasingly important role in consumer food choice behavior and the common perception that cereal products are often high in sugar [9,10], this study also examined how price fairness affects consumers’ assessment when selecting healthier cereal options. The findings highlight the role of price in shaping consumer behavior, particularly in the context of choosing healthier alternatives within the cereal market. Furthermore, the results of the median split analysis implied that consumers perceiving low price fairness are more sensitive to building positive attitudes through the nutritional value of cereal products. It can be inferred that consumers who perceive expensiveness (lower level of price fairness) to the cereal products expect a higher level of nutritional value in the case of cereal products, implying that high price segment could take care of food healthiness more for cereal products.

## 6. Conclusions

### 6.1. Theoretical and Managerial Implications

This work explored the relationship between nutritional value, consumer attitude, and repurchase intention among cereal product consumers, yielding statistically significant results. This research focused on the North American market and data collection was implemented via an online survey. Building on prior research that identifies the nutritional aspects as a potential weakness of cereal products [9,10], this study sought to examine the impact of these nutritional factors on consumer behavior. Furthermore, the research empirically confirmed the significant moderating effect of price fairness on the relationship between nutritional value and consumer attitude. By investigating the determinants of repurchase intention, attitude, nutritional value, and price fairness in the context of cereal products, this study makes a valuable contribution to the literature. The findings provided external validation of the positive relationship between attitude and repurchase intention, which aligned with previous studies in this area [3,38]. Moreover, the results supported the influence of nutritional value on both attitude [44] and repurchase intention [7], reinforcing the existing research in the field. In this regard, the study extended the application of these findings from the broader food consumer behavior domain to cereal product consumers, thereby contributing theoretically. A key contribution of this study was the identification of price fairness as a significant moderating factor in the relationship between nutritional value and consumer attitude. This insight enhanced our understanding of consumer behavior in the cereal product market. By emphasizing the role of price fairness, the study also added to the literature on the low-involvement theory, demonstrating its relevance to cereal product consumption behavior. It highlighted how price perceptions influenced consumer attitudes and provided a more nuanced understanding of how low-involvement products, such as cereals, were shaped by both nutritional factors and price fairness. Therefore, this work extended the low-involvement theory into the context of cereal product consumption, further validating its explanatory power and offering worthwhile theoretical contributions.

In terms of managerial implications, this research provides several actionable recommendations. First, cereal product managers might be able to prioritize investments in the nutritional aspects of their products, as the findings noted that nutritional value plays a crucial role in shaping both consumer attitudes and repurchase intentions. To effectively communicate with consumers, managers might be able to consider emphasizing health-related information, as this could positively influence consumer attitudes and repurchase intentions, potentially boosting sales. Moreover, integrating Environmental, Social, and Governance (ESG) initiatives into marketing strategies may enhance the brand’s reputation and foster more favorable consumer attitudes.

Next, it may be beneficial for managers to tailor their marketing strategies according to both price sensitivity and nutritional value, as consumer preferences can vary based on their perceptions of price fairness. Given the sensitivity to price in the cereal market, managers should be cautious with price increases or fluctuations, as such changes may lead to negative consumer reactions and impact long-term sales. Finally, emphasizing competitive pricing through strategies like offering discounts or highlighting more attractive prices compared to competitors could help improve consumers’ perceptions of price fairness and encourage positive responses.

### 6.2. Limitations and Directions for Future Research

This study has limitations. First, this study focused on four key attributes influencing consumer behavior in the cereal market. However, future research could expand on these findings by exploring additional factors such as brand loyalty, taste preferences, convenience, labeling and packaging, and environmental considerations, offering a more comprehensive understanding of consumer behavior. Second, the study relied solely on a survey-based data collection method. To enrich insights into consumer perceptions and decision-making processes regarding cereal products, future research might incorporate other methodologies, such as interviews or experimental designs. Third, the survey responses were collected via an online questionnaire, with a higher proportion of female respondents. This gender imbalance could have influenced the results, suggesting that future research might be able to ensure a more balanced representation by addressing gender aspects in the sampling process. Furthermore, while this work focused on the North American market, future research could include consumers from diverse cultural backgrounds to improve the generalizability of the findings. Another limitation of this study is that it approached cereal products as a broad category without considering specific product types. Future research could examine different types of cereal products to gain a deeper understanding of consumer behavior. Lastly, conducting qualitative studies to explore consumers’ perceptions of cereal products would provide valuable insights and further enhance the understanding of consumer preferences in this market.

## Figures and Tables

**Table 1:** Depiction of measurement.

Construct	Code	Item	Reference
Price fairness	P1	The price of cereal products is fair.	Carmona et al. [46]; Grashius & Ye [15]
	P2	The price of cereal products is rational.	
	P3	The price of cereal products is reasonable.	
	P4	The price of cereal products is affordable.	
Nutritional value	H1	The nutritional value of cereal products is adequate.	Hati et al. [5]; Lin et al. [7]
	H2	Cereal products are nutritionally balanced.	
	H3	The nutrition of cereal products is good.	
	H4	The nutritional quality of cereal products is appropriate.	
Attitude	A1	Cereal products are (negative-positive)	Jung et al. [13]; Asti et al. [3]
	A2	Cereal products are (bad-good)	
	A3	Cereal products are (worthless-worthwhile)	
	A4	Cereal products are (unfavorable-favorable)	
Repurchase intention	R1	I am going to purchase cereal products again.	Asti et al. [3]; Sun & Moon [4]
	R2	I intend to buy cereal products again.	
	R3	I am going to repurchase cereal products.	
	R4	I have a repurchase intention of cereal products.	

**Table 2:** Demographic information (N = 414).

Item	Frequency	Percentage
Male	124	30.0
Female	290	70.0
20–29 years old	68	16.4
30–39 years old	129	31.2
40–49 years old	150	36.2
50–59 years old	56	13.5
Older than 60 years old	11	2.7
Weekly eating frequency		
Less than 1 time	91	22.0
1–2 times	143	34.5
3–6 times	132	31.9
Everyday	48	11.6
Terminal academic degree		
Less than a bachelor’s degree	159	38.4
Bachelor degree	174	42.0
Graduate degree	81	19.6
Monthly household income		
Less than USD 2500	113	27.3
Between USD 2500 and USD 4999	162	39.1
Between USD 5000 and USD 7499	58	14.0
Between USD 7500 and USD 9999	32	7.7
More than USD 10,000	49	11.8

**Table 3:** Results of factor analysis.

Construct	Code	Loading	Mean (SD)	Cronbach’s α	Eigenvalue	Explained Variance (%)
Price fairness	P1	0.897	3.04 (1.05)	0.946	1.848	11.547
	P2	0.886				
	P3	0.936				
	P4	0.885				
Nutritional value	H1	0.855	3.18 (1.04)	0.955	2.835	17.720
	H2	0.892				
	H3	0.895				
	H4	0.857				
Attitude	A1	0.827	4.23 (0.82)	0.910	1.057	6.603
	A2	0.811				
	A3	0.731				
	A4	0.776				
Repurchase intention	R1	0.885	4.38 (0.88)	0.959	7.988	49.926
	R2	0.902				
	R3	0.894				
	R4	0.893				

Note: SD stands for standard deviation; the unit of explained variance is percent, total variance explained: 85.291; Kaiser–Meyer–Olkin measure (KMO) of sampling adequacy: 0.909; Bartlett’s test of sphericity Approx. Chi-Square: 7122.568 (*p* < 0.01).

**Table 4:** Correlation matrix.

	1	2	3	4
1. Repurchase intention	1			
2. Attitude	0.654 *	1		
3. Nutritional value	0.417 *	0.554 *	1	
4. Price fairness	0.264 *	0.322 *	0.425 *	1

Note: * *p* < 0.05.

**Table 5:** Results of hypotheses testing: the moderating effect of price fairness.

	Model 1a Attitude	Model 1b Attitude	Model 2a Repurchase Intention	Model 2b Repurchase Intention
	β (t value)	β (t value)	β (t value)	β (t value)
Constant	2.020 (6.99) **	2.087 (6.92) **	1.419 (8.28) **	1.356 (6.30) **
Nutritional value	1.070 (6.85) **	0.483 (5.50) **	0.066 (1.76) *	0.035 (0.96)
Price fairness	0.393 (3.27) **	0.252 (2.68) **		
Interaction	−0.071 (−2.59) **	−0.052 (−1.98) **		
Attitude			0.350 (13.65) **	0.565 (11.47) **
Sex		0.019 (0.27)		0.137 (1.96) *
Age		0.002 (0.08)		0.032 (1.01)
Weekly eating frequency		0.233 (6.37) **.		0.188 (4.89) **
Terminal academic degree		−0.120 (−2.73) **		−0.028 (−0.64)
Monthly household income		0.010 (0.41)		−0.019 (−0.77)
F-value	66.51 **	34.75 **	24.87 *	52.30 **
R^2^	0.3274	0.4071	0.1077	0.4742
Conditional effect of the focal predictor				
Price fairness				
2.00	0.474 (10.57) **	0.379 (8.37) **		
3.00	0.402 (11.37) **	0.326 (9.08) **		
4.00	0.330 (7.35) **	0.274 (6.25) **		
Index of mediated moderation	Index	Index		
	−0.0467 **	−0.0296 **		

Note: * *p* < 0.1 ** *p* < 0.05, interaction: nutritional value × price fairness in model 1a (test of interaction: F = 6.75 **) and model 1b (test of interaction: F = 3.94 **).

## Data Availability

The data presented in this study are available upon request from the corresponding author. The data are not publicly available due to privacy concerns.

## References

[1] T4 (2024). Cereal Companies Market Share. https://www.t4.ai/industries/cereal-companies-market-share.

[2] Grand View Research (2024). Breakfast Cereal Market Size, Share & Trends Analysis Report by Product (Hot Cereals, and Ready-to-Eat), By Distribution Channel. https://www.grandviewresearch.com/industry-analysis/breakfast-cereals-market.

[3] Asti W., Handayani P., Azzahro F. (2021). Influence of trust, perceived value, and attitude on customers’ repurchase intention for e-grocery. J. Food Prod. Mark..

[4] Sun K.A., Moon J. (2024). Relationships between Psychological Risk, Brand Trust, and Repurchase Intentions of Bottled Water: The Moderating Effect of Eco-Friendly Packaging. Sustainability.

[5] Hati S., Zulianti I., Achyar A., Safira A. (2021). Perceptions of nutritional value, sensory appeal, and price influencing customer intention to purchase frozen beef: Evidence from Indonesia. Meat Sci..

[6] Sobaih A., Algezawy M., Elshaer I.A. (2023). Adopting an extended theory of planned behaviour to examine buying intention and behaviour of nutrition-labelled menu for healthy food choices in quick service restaurants: Does the culture of consumers really matter?. Int. J. Env. Res. Public Health.

[7] Lin J., Li T., Guo J. (2021). Factors influencing consumers’ continuous purchase intention on fresh food e-commerce platforms: An organic foods-centric empirical investigation. Electron. Commer. Res. Appl..

[8] Stelick A., Sogari G., Rodolfi M., Dando R., Paciulli M. (2021). Impact of sustainability and nutritional messaging on Italian consumers’ purchase intent of cereal bars made with brewery spent grains. J. Food Sci..

[9] LoDolce M.E., Harris J.L., Schwartz M.B. (2013). Sugar as part of a balanced breakfast? What cereal advertisements teach children about healthy eating. J. Health Comm..

[10] Prada M., Saraiva M., Viegas C., Cavalheiro B., Garrido M. (2021). Examining the relationship between sugar content, packaging features, and food claims of breakfast cereals. Nutrients.

[11] Anshu K., Gaur L., Singh G. (2022). Impact of customer experience on attitude and repurchase intention in online grocery retailing: A moderation mechanism of value Co-creation. J. Retail. Consum. Serv..

[12] Amoroso D., Ackaradejruangsri P. (2017). How consumer attitudes improve repurchase intention. Int. J. E-Serv. Mob. Appl..

[13] Jung H.J., Choi Y., Oh K. (2020). Influencing factors of Chinese consumers’ purchase intention to sustainable apparel products: Exploring consumer “attitude–behavioral intention” gap. Sustainability.

[14] Severt K., Shin Y., Chen H., DiPietro R.B. (2022). Measuring the relationships between corporate social responsibility, perceived quality, price fairness, satisfaction, and conative loyalty in the context of local food restaurants. Int. J. Hosp. Tour. Adm..

[15] Grashius J., Ye S. (2023). Farmer-owned brand purchases: The importance of label comprehension and price fairness perception. Study Agric. Econ..

[16] Hride F.T., Ferdousi F., Jasimuddin S. (2022). Linking perceived price fairness, customer satisfaction, trust, and loyalty: A structural equation modeling of Facebook-based e-commerce in Bangladesh. Glob. Bus. Organ. Excell..

[17] Samoggia A., Grillini G., Del Prete M. (2021). Price fairness of processed tomato agro-food chain: The Italian consumers’ perception perspective. Food.

[18] Drichoutis A.C., Lazaridis P., Nayga R. (2007). An assessment of product class involvement in food-purchasing behavior. Eur. J. Mark..

[19] Bissinger K. (2019). Price fairness: Two-stage comparison of conventional and fairtrade prices. J. Int. Consum. Mark..

[20] Mitchell V., Boustani P. (1992). Consumer risk perceptions in the breakfast cereal market. Br. Food J..

[21] Adhikari A. (2019). Consumer behavior in low involvement product purchase: A stochastic model. Theor. Econ. Lett..

[22] Lim C., Hong K., Wong S., Yee L. (2021). Interaction Effect of Country of Manufacture and Brand Awareness on Malaysian Young Adults Purchase Intention of Low Involvement Product. Mark. Sci. Res. Organ..

[23] Lim W., Guzmán F. (2022). How does promotion mix affect brand equity? Insights from a mixed-methods study of low involvement products. J. Bus. Res..

[24] Andreosso-O’Callaghan B., Zolin M. (2010). Long-term cereal price changes: How important is the speculative element?. Transit. Stud. Rev..

[25] Cedrez C.B., Chamberlin J., Hijmans R.J. (2020). Seasonal, annual, and spatial variation in cereal prices in Sub-Saharan Africa. Glob. Food Secur..

[26] Singh S., Alok S. (2022). Drivers of repurchase intention of organic food in India: Role of perceived consumer social responsibility, price, value, and quality. J. Int. Food Agribus. Mark..

[27] Xiao M., Razzaq A., Qing P., Phromphithakkul W., Thinakaran R., Alnafissa M. (2024). Reducing food waste and promoting sustainable consumption: The role of message framing and controllability attributions in ugly produce marketing. Front. Sustain. Food Syst..

[28] Biondi B., Camanzi L. (2020). Nutrition, hedonic or environmental? The effect of front-of-pack messages on consumers’ perception and purchase intention of a novel food product with multiple attributes. Food Res. Int..

[29] Ali B. (2021). Consumer attitudes towards healthy and organic food in the Kurdistan region of Iraq. Manag. Sci. Lett..

[30] Sadiq M., Rajeswari B., Ansari L., Kirmani M. (2021). The role of food eating values and exploratory behaviour traits in predicting intention to consume organic foods: An extended planned behaviour approach. J. Retail. Consum. Serv..

[31] Zebregs S., van den Putte B., Neijens P., de Graaf A. (2015). The differential impact of statistical and narrative evidence on beliefs, attitude, and intention: A meta-analysis. Health Comm..

[32] Ma C., Chang H.P. (2022). The effect of novel and environmentally friendly foods on consumer attitude and behavior: A value-attitude-behavioral model. Foods.

[33] Zhang B., Zhang Y., Zhou P. (2021). Consumer attitude towards sustainability of fast fashion products in the UK. Sustainability.

[34] Afrizal D., Wallang M. (2021). Attitude on intention to use e-government in Indonesia. Indones. J. Electr. Eng. Comput. Sci..

[35] Chawla D., Joshi H. (2019). Consumer attitude and intention to adopt mobile wallet in India—An empirical study. Int. J. Bank Mark..

[36] Xu A., Wei C., Zheng M., Sun L., Tang D. (2022). Influence of perceived value on repurchase intention of green agricultural products: From the perspective of multi-group analysis. Sustainability.

[37] Nazri N., Ngah A., Rahi S., Gabarre S., Long F., Rashid A. (2024). Bon appetit! Factors influencing consumers’ repurchase intention at food trucks in Malaysia. J. Foodserv. Bus. Res..

[38] Hussain K., Fayyaz M., Shamim A., Abbasi A., Malik S., Abid M. (2024). Attitude, repurchase intention and brand loyalty toward halal cosmetics. J. Islam. Mark..

[39] Moon J., Ji Y. (2023). Structural Relationship between Taste, Price Fairness, and Repurchase Intention of Fast Food: Moderating Effect of Healthiness. Glob. Bus. Financ. Rev..

[40] Loh Z., Hassan S.H. (2022). Consumers’ attitudes, perceived risks and perceived benefits towards repurchase intention of food truck products. Br. Food J..

[41] Riaz H., Davidaviciene V., Ahmed H., Meidute-Kavaliauskiene I. (2022). Optimizing customer repurchase intention through cognitive and affective experience: An insight of food delivery applications. Sustainability.

[42] Park C., Namkung Y. (2024). Consumer Values, Attitudes, and Behavior towards Plant-Based Alternatives. Foods.

[43] Thanki H., Shah S., Oza A., Vizureanu P., Burduhos-Nergis D. (2022). Sustainable consumption: Will they buy it again? Factors influencing the intention to repurchase organic food grain. Foods.

[44] Su W., Zhang Y., Li S., Sheng J. (2023). Consumers’ Preferences and Attitudes towards Plant-Based Milk. Foods.

[45] Baiano A. (2022). 3D printed foods: A comprehensive review on technologies, nutritional value, safety, consumer attitude, regulatory framework, and economic and sustainability issues. Food Rev. Int..

[46] Carmona I., Griffith D.M., Aguirre I. (2021). Understanding the factors limiting organic consumption: The effect of marketing channel on produce price, availability, and price fairness. Org. Agric..

[47] Harris G. (1987). The implications of low-involvement theory for advertising effectiveness. Int. J. Adv..

[48] Purcini G., Medeiros Barretta L., Ferreira L., Lourenção M. (2024). Analyzing the impact of country-of-origin, geographical indication and wine world on low-involvement generation Z potential consumers’ attitudes toward wine ads. Int. J. Wine Bus. Res..

[49] Park S., Wei X., Lee H. (2024). Revisiting the elaboration likelihood model in the context of a virtual influencer: A comparison between high-and low-involvement products. J. Consum. Behav..

[50] Ugalde C., Küster I., Vila N. (2024). Brand attachment: The moderating effect of high and low involvement products. J. Consum. Sci..

[51] Lou C., Xie Q. (2021). Something social, something entertaining? How digital content marketing augments consumer experience and brand loyalty. Int. J. Adv..

[52] Jiang K., Zheng J., Luo S. (2024). Green power of virtual influencer: The role of virtual influencer image, emotional appeal, and product involvement. J. Retail. Consum. Serv..

[53] Yun S., Jun S., Kim J.W. (2024). The impact of high arousal and displeasure on online review helpfulness: Exploring the moderating role of product involvement. Electron. Commer. Res. Appl..

[54] Grashuis J. (2024). Price fairness in the online food delivery industry: A structural equation model of consumer perceptions. J. Food Bus. Res..

[55] Artuğer S., Sayın K., Kilinç Şahïn S. (2024). The effect of social servicescape on price fairness and customer trust: A study in coffee shops. Br. Food J..

[56] Lucian S. (2024). Examining the work need satisfaction scale in the online platform gig work environment: A structural and contextual analysis. Jurnalul Pract. Comunitare Pozitive.

[57] Mayer A., Ihl A., Grabl S., Strunk K., Fiedler M. (2024). A silver lining for the excluded: Exploring experiences that micro-task crowdsourcing affords workers with impaired work access. Inf. Syst. J..

[58] Hair J., Anderson R., Babin B., Black W. (2010). Multivariate Data Analysis: A Global Perspective.

[59] Hayes A. (2017). Introduction to Mediation, Moderation, and Conditional Process Analysis: A Regression-Based Approach.

